# Using Curriculum Mapping as a Tool to Match Student Learning Outcomes and Social Studies Curricula

**DOI:** 10.3389/fpsyg.2022.850264

**Published:** 2022-08-18

**Authors:** Monday U. Okojie, Mert Bastas, Fatma Miralay

**Affiliations:** Faculty of Education, Near East University, Nicosia, Cyprus

**Keywords:** curriculum mapping, COVID-19 pandemic, gap, redundancy, virtual education

## Abstract

The interest in program- and colleges of education- level evaluation and alignment of student learning outcomes to course content has been increasing over the past several decades. Curriculum mapping establishes the links between content and expected student learning outcomes. Curriculum map is an overview of what is taking place in the classroom; and it includes evaluation tools and activities. Social Studies Department, Federal Capital Territory (FCT) College of Education Zuba, Abuja, recently completed an accreditation exercise by National Commission for Colleges of Education Abuja, Nigeria. The audit reported that there was no match between the student learning outcomes and Social Studies curricula. The purpose of this paper was to align the Nigeria Certificate in Education (NCE) (Social Studies) minimum standards with student learning outcomes to determine gaps and redundancies. The paper also looked at how virtual education enhances curriculum mapping during COVID-19 pandemic. Minimum standards learning outcomes were modified from existing learning outcomes to better align with college learning outcomes and the Social Studies Core and Elective Competencies. All NCE Social Studies courses were mapped to the Social Studies Core and Elective Competencies and assessed to determine the gaps and redundancies. The study used the documentary research method. The purposeful sampling strategy was used to select the research site. Potential gaps were defined as coverage for each competency in about ≤20% of the courses and potential redundancies was considered as coverage of ≥80% of the courses. The mapping exercise revealed gaps; and no redundancies in course content. The findings of the mapping exercises should be used to improve the content provided to NCE Social Studies students at FCT College of Education Zuba, with the overall objective of enhancing the quality of the education provided to those students and helping them to be better students that are prepared for a successful career in Social Studies.

## Background and Rationale

Harden (2001) defines “curriculum as complex combination of educational approaches, course content, learning attributes, educational experiences, evaluation, the educational environment and the individual student’ learning style, personal time table and program of study” (p. 123). He added that curricula can be intended, designed/planned, communicated, enacted and evaluated from the perspective of an educator and, from a student’s perspective, experienced and learnt. Mapping of curriculum is developed to “explore how to impart knowledge with skills (e.g., critical thinking, creative thinking, teamwork, and meta-cognitive skills) and attitudes and values (e.g., curiosity, respect empathy)” (The Organization for Economic Co-operation and Development [OECD], 2016, p. 1).

To provide association amongst drafted curriculum and learned curriculum, English in Archambault and Masunaga (2015) initiated the procedure of mapping curriculum that explains what is truly taught, period of time taught, association amongst what is being taught and the district’s testing program. Therefore, mapping curriculum is a method of analyzing the program and courses within curriculum to clarify curriculum frameworks and relationships, to gain insight into how students perceive their discipline and to increase awareness of curriculum content (Archambault and Masunaga, 2015). With the increased demand for education reform, there is a need for educators to provide a curriculum that promotes increased skills and competency attainment for graduates as opposed to more traditional curriculum design methods (Neville-Norton and Cantwell, 2019).

During the academic year 2015–2016, the Department of Social Studies, FCT COE Zuba obtained educational accreditation to upgrade the National Commission for colleges of Education (NCCE) Accreditation (Ogunrinade, 2013). Accreditation showed that there was no process to match the proposed curriculum with the actual curriculum and assessments (NCCE, 2016). The success of the student can be strongly influenced by differences and redundancies in the curriculum. It was necessary to pay attention to the issue of not providing a plan to match the actual curriculum with the planned curriculum because misalignment of the curriculum could result in discrepancies and redundancies in both skills and content between and within grades (NCCE, 2016). Hence, there was the need to look at the use of curriculum mapping as a tool to match student learning outcomes and Social Studies curricula of Department of Social Studies.

The Social Studies department conducted program map practice, supervised by correspondent researcher to meet college assessment criteria and results.

## Pedagogical Framework

### Curriculum Mapping Process

Curriculum mapping is a process of developing a visual map of all courses in the curriculum and evaluating course content to determine if any gaps or excessive overlap exist and to ensure all courses meet curriculum learning outcomes (Harden, 2001). He added that to find out the level of association, every course is assessed in agreements of the learning outcomes of the instruction, benchmarks for association and measure of inclusion. If finished, the picture of the course of study permits learners, teachers and school managers to comprehend how every program suits the program and which courses are dependent on each other (Meij and Merx, 2018). Curriculum mapping can also define and address gaps and redundancies in coverage (Liu et al., 2010).

Curriculum mapping is another model of curriculum development that also places great emphasis on cooperation among educators. Udelhofen (2005) says instructional mapping is the system that instructors records the instruction that belong to them, exchange, review their curricula on discrepancies, differences, setbacks including current experiences, and create cohesive, reliable instruction at all places of learning which relatively matched with benchmarks. Jacobs (2004) suggests that curriculum mapping is useful to recognize the vertical and horizontal alignment of learning outcomes within a course and within the system as a whole. Curriculum mapping can be a tool to help create a high-quality curriculum based on that knowledge.

While Colleges of Education in Nigeria increasingly pursue quantitative data to track departmental and college results, curriculum mapping will progressively be needed from every occupation to produce analytical measurement of instruction as the pace of student through the course of study as well as assessing the achievement of the program. The department of correspondent author, Social Studies for instance, has college assessment and National Commission for Colleges of Education (NCCE) accreditation requirements to meet (Abdulmalik, 2019). In fact, FCT College of Education is encouraging curriculum mapping for every occupation to initiates measurable impact learning and gets how minimum standards subjects associate with those attributes.

### The Meaning of Social Studies

Social Studies deals with man in his environment using science and technology as a means of solving the problems of his environment (Comparative Education Study and Adaptation Centre [CESAC], 1984). The environment comprises of physical and social environment. It is because man lives in groups or societies that we say he lives in a social environment. Similarly, man lives in a physical environment because he lives in a territory and uses what he can get from that territory. Meanwhile, we should remember that Social Studies is concerned with the way man lives in and interacts with his social and physical environments and how science and technology help him to live well in those environments. Generally we use the word person or human beings when it is not important to say whether you are talking about a man or a woman. In the context of this paper, man is regarded as person or human beings to avoid gender disparity. This is because many people no longer use man to mean ‘men and women in general’ because it gives the impression that woman is not included.

Therefore, Social Studies is a program of study which a society uses to instill in students the knowledge, skills, attitudes, and actions it considers important concerning the relationships human beings have with each other, their world, and themselves (Kissock, 1981). The concern for the teaching of Social Studies in Nigeria originated out of the need to make education relevant to the needs of the society and to prepare human beings for a useful life wherever they find themselves. Social studies is not only concerned about the development of the cognitive aspect, the subject also intends to inculcate in the learner those values and skills that will enable them to function effectively in their society. Social Studies integrates ideas, knowledge, information and concepts from the Social Sciences and other disciplines to develop the skills and values for effective citizenship (Falade, 2008).

Social Studies is one of the subjects in the Nigerian schools which helps the educational system work toward achieving set national objectives. The general objectives of Social Studies education according to Nzeribe (2002) may be listed as follows:

1. To give human beings adequate information and knowledge about their society and the wider world.
2. To create in human beings an awareness and appreciation of the benefits and results of scientific and technological discoveries and intentions and make them see how these affect their everyday life.
3. To help human beings develop their intellect skills, abilities and competencies and promote in them the spirit of enquiry, discovery, thinking and curiosity which act as a spur to further investigation.
4. To make human beings know what the society expects of its members so that they will be able to judge his actions as well as those of others.
5. To familiarize human beings with the norms of their society, and thus socialize them in accordance with such norms. This will enable them improve and perpetuate their society.
6. To help human beings become a good citizen and develop the necessary values and attitude needed in democracy.
7. To create in human beings an awareness and appreciation that community life in any human society is based on co-operation and inter-dependence at all levels right from the family to the international level.
8. To help human beings develop proper value judgment and ability to criticize and select, and place events in their proper perspectives.
9. To enable human beings develop psychomotor skills involving locomotion and non-locomotion skills, manipulative and creative skills, perceptual and physical abilities.
10. To help human beings develop valuable and socially acceptable concepts, ideas and philosophy of life.

### How Virtual Education Enhances Curriculum Mapping During COVID-19 Pandemic

Following significant school closures enforced as part of public health efforts to limit the circulation of corona virus (United Nations Educational Scientific and Cultural Organization [UNESCO], 2020), education systems around the world are confronting an unprecedented challenge. According to UNESCO, the corona virus outbreak would have affected over 1.37 billion children and teachers by April 2020, including at work (Corlatean, 2020). In other words, 80% of all students in a global educational system are at risk of falling behind intellectually in the next years, risking future growth rates and income from their fields of activity (Wilkerson, 2020).

Education is one of the social arenas that have faced the most significant difficulties without being adequately equipped. For pupils, students, and teachers, the pandemic resulted in the closure of schools and universities, resulting in significant changes in the educational process in a short period of time, as well as a significant loss of time allocated to learning by many students, with potentially negative consequences for their educational progression and profession (Corlatean, 2020). Many governments encouraged schools to shift from traditional to virtual education and online learning in the aftermath of the corona virus spread, when kids were barred from going to school and face-to-face schooling discontinued (Rogers and Sabarwal, 2020).

Virtual education (online education and learning) is a type of online learning that distinguishes itself by separating the student and the instructor (Cavanaugh et al., 2004). Virtual education and internet based instructional methods could be actively promoted to complement teaching if they are supported by sufficient professional settings and assistance, according to the data (Kundu, 2020). Education program operators must explain to learners, faculty, and external regulators the program’s curriculum and how it matches with desirable results. A virtual education program regurgitates and map components from an existing program related to a given topic to provide explicit visual aids by marking each time the topic is addressed, performed, and examined (Bateman et al., 2015). Particular aspects remain in existing current courses, and there is no disturbance to the school curricula, but there is an improvement in terms of demonstrating relationships inside a topic in a transparent manner.

Curriculum revision to incorporate new information could basically entail adding more teaching and learning process; nevertheless, Social Studies curricula are probably sufficiently overburdened, so adding more material may be unnecessary. As a result, a different way to simplifying “adding additional curricular material” could be a thorough examination of the previous curricula with the goal of recognizing where larger abilities are already present subtly within the program (Bateman et al., 2015). These periphery topics can now be “road-mapped” into a cross-cutting virtual educational program, allowing the providers to pinpoint and visibly exhibit the full scope of the curricula. We agree that this kind of curricula topic signposting for learners or faculty is suitable. It can also be a good way to show regulatory agencies that you’re complying with new competency criteria with little or no disturbance to your present curriculum.

Every program has a designated leader who is in charge of all aspects of the program, along with the design of learning outcomes and regulatory compliance course features a guide that identifies the goals, learning outcomes, schedule of study, accompanying reading, and a description of how the subject will be tested, facilitating student involvement (Harden et al., 1999). The notion of virtual education courses will be designed to illustrate where cross-curricula topics are taught and assessed. By identifying each time it occurs all through the curriculum, a virtual education course gives obvious signposting to parts of teaching and evaluation in a certain periphery topic. It is critical to note that establishing a virtual education program does not imply that the topic is not directly delivered; rather, it is taught in several programs and incorporated into core teaching to the point where it becomes indivisible from other competence or field (Bateman et al., 2015). Every virtual education program has genuine content and takes the form of lectures, seminars, and practical sessions, but each activity is contained within a core course in the program. The goal is to create a thorough course guide for each virtual education course that follows the same design as a conventional classroom.

Generating a virtual education program that incorporates exercise from various sessions of a curriculum is difficult for one person to accomplish because there is a major risk of missing contributory exercises; a working group of academic leads for courses that emerge to have similarity with regard to the virtual education course topic will be formed. The scope and outline goals of the virtual education program will be decided at the first working group meeting so that each group member is secure in their knowledge of the goal, and a basic understanding of available contents will be developed. Individual members of the team will be given the task of creating inspiring learning abilities for the virtual education program. These will take into consideration regulatory standards (General Dental Council, 2012), the Graduate Skills Framework of the FCT College of Education, and our own ambitions for Social Studies courses, which go above and beyond the minimum needs set by external stakeholders.

Once the learning outcomes from each of the above sources have been found, areas of duplication will be integrated and transcribed into course learning outcomes, which will then be organized into sub-topics and correctly categorized to aid mapping and blueprinting. The working group will identify activities that are already included in core courses and will develop the virtual education course’s program of study in order to create a planned schedule specifying when they occur in the overall curriculum. Lectures, seminars, individual or group projects, and other exercises that contribute will be included. When a possible virtual education course component is discovered, a brief description of the exercise, where it occurs (core course and stages), and how it contributes to virtual education course learning outcomes will be offered.

The introduction and goals of virtual education will be completed at this point, with a quick description of the exercises and learning outcomes. In addition to the reading previously mentioned in the core course, the working group will offer related reading and other resources. Learners will be referred to these particular core course guides as well as the supporting information in the virtual education course guide in order to avoid duplication. Where the theme may be assessed, both formative and summative, will be taken into account, and this data will be supplied.

The final course map will then be distributed to all program course leaders for input and data on educational aspects that may add to the virtual education course’s subject. The procedure of soliciting course leaders’ opinions, in addition to improving and authenticating content, develops awareness and a sense of control of the new virtual education course, as explained by Gale and Grant (1997). The information will then be updated as needed.

A program leader will be assigned at this stage. This individual will be in charge of raising awareness of the program, keeping the program guide up to date, conducting course reviews as part of our organized curriculum review program, and leading the preparation of evaluation for the topic addressed. In the future, the course leader will be the point of contact for any questions about the course material from both faculty and learners. All faculty and learners will then have access to the completed virtual education course guide.

## Learning Environment

The Federal Capital Territory College of Education is located in the Federal Capital Territory and serves a population of 3,467,123 people. The College has a history of strong community support and a reputation for high standards and performance. The Social Studies Department has 1,875 students enrolled in Nigeria Certificate in Education (NCE) One (1), Two (2), and Three (3) courses in suitable lecture halls at the time of inquiry. The academic year is divided into two semesters, with lectures lasting 1, 2, or 3 h each. The student body is entirely made up of Black Africans. FCT College of Education students come from many cultural backgrounds and speak a variety of languages.

In total of 45.5 percent of the department of Social Studies’ 11 licensed teachers have a master’s degree, while 54.5 percent have a doctorate. Teachers are grouped by minimal criteria areas, with the Social Studies department being led by a Departmental Head. Formal professional interactions between teachers occur at whole-school staff meetings, departmental meetings, and professional development days, which are held on a semester-by-semester basis. End-of-semester breaks have been implemented to provide academics greater time to engage in professional activities and collaborate.

The teaching staff and administration of Social Studies appear to seek to provide students with the best educational opportunities available, as evidenced by the College purpose statement:

“The mission of FCT College of Education is to provide the teachers requirements for both the primary and post-primary schools within the Federal Capital Territory and its environs.”

As a result, the College places a premium on objectivity in their minimal standards in order to assure academic achievement for students while also fostering positive relationships with students and their parents. The College’s high academic expectations have resulted in students’ good performance on end-of-semester exams and high scores year after year, high graduation rates year after year, and a large number of graduates entering the labor market and universities for further studies. Students’ hard work, instructor professionalism, family involvement, and good administrative leadership, according to the teachers, contributed to the school’s success. The department of Social Studies has a culture that emphasizes the value of collegiality and collaboration, teacher and student growth, and the college’s collective and individual achievement.

## Learning Objectives

The learning objectives of the research were to:

a. determine the distribution of learning outcomes as expressed in the Social Studies program course organization;
b. find out what extend the learning outcomes of Social Studies are consistent with the curricula expected (Social Studies curricula);
c. detect the discrepancies, redundancies and changes required to match learning outcomes and curricula for Social Studies; and
d. unveil how virtual education enhances curriculum mapping during COVID-19 pandemic.

## Significant Statement

This study discovered the potentials that can be beneficial for parents, schools, learners, teachers, government, Ministry of Education, curriculum planners, school managers, and NGOs in education sector, etc. This study will help the researchers to uncover the critical areas of curriculum alignment, gaps, duplications, discrepancies and benefits with programs that many researchers were not able to explore in Nigeria educational sector when done in a collaborative format. Thus, a new theory on student academic performance may be arrived.

## Pedagogical Format

### Creating Curriculum Learning Outcomes

The 1st stage in drafting the process of a program is to create assessable attributes (Palomba and Banta, 1999). Baumann and Harvey (2012) refer to curriculum alignment as an implicit correlation between the content of the course, the learning tasks, the teaching methods and the assessment of a subject in order to achieve the expected learning outcomes. Lawson et al. (2015) define attributes as abilities learners acquire when the program is completed. In addition, program attributes contains insight including abilities not studied in a particular subject, but *via* encounters and organization of insight and abilities from different subjects into a full disciplined (Joyner Melito, 2016a) and these program attributes are useful for production of a unified course of study (Hubball and Burt, 2007).

Attributes subsisted in Social Studies Department preceding the commencement of map practice. These attributes were ambiguous and not recorded in best acceptable shape for attributes. Consequently, these attributes were padded for clarity and measurable (Joyner Melito, 2016a). During the consideration of the development of Social Studies Nigeria Certificate in Education (NCE) learning outcomes, consideration was given to College of Education Zuba, Abuja, and National Commission for Colleges of Education Abuja audit qualification for learner insight and abilities, i.e., Social Studies required skills and attributes. Attributes were further outlined to be measurable for simple of forthcoming program evaluation (Joyner Melito, 2016a). The rewritten student learning outcomes is shown in Table 1 while Course title and Social Studies Core Competency category and Electives is shown in Table 2.

**TABLE 1:** Objectives of NCE social studies from the minimum standards.

Objective codes	Objectives of NCE Social Studies
SOS 111	This course is intended to expose students to the philosophy and basic characteristics of Social Studies Education.
SOS 112	The idea of man as a social being and why he lives as group is the focus of this course.
SOS 113	The course is designed to uplift the knowledge of students on the physical environment, how it influences and how man through his numerous activities influences the physical environment.
SOS 121	The course introduces students to the NERDC National Curriculum of Social Studies for Basic Education 7–9.
SOS 122	The students are taken through the evolution of the Nigerian National and to appraise the cultural diversities of our nation.
SOS 123	This course is designed to expose students to the origin and nature of man.
SOS 124	The focus of this course is to introduce the learners to the major economic activities within the Nigerian State.
SOS 125	This course intends to expose the learners to the rudiments of governance in human society.
SOS 211	This course aims at exposing students to the concepts of the Nigeria Political Life in relation to the general provisions of the Nigerian Constitution.
SOS 212	This course aims at exposing students to practical application of NERDC National Curriculum for Social Studies.
SOS 213	This course aims at exposing students to principles of research and statistical methods for effective research work in Social Studies.
SOS 214	The course will afford the students the opportunity to visit both far and near environment in terms of educative interest in Social Studies.
SOS 221	The course is designed to expose students to basic concepts of National Development.
SOS 222	This course introduces students to some concepts of citizenship education.
SOS 223	This course focuses on the institutions that provide public utilities and the factors and processes of social change.
SOS 224	The course seeks to describe the element of laws, rules, regulations, ordinances, edits, decrees, norms and mores as it affect the modern society.
SOS 225	The course seeks to expose students to various means of transportation and communication.
SOS 321	The course focuses attention on population and Family Life Education.
SOS 322	The course focuses on the principles of International Relations and Nigeria’s Foreign Policy.
SOS 323	The course is designed to expose students to the structure, functions and problems of different social institutions in Nigeria.
SOS 324	This course aims at exposing students to the concepts of globalization in relation to the impact it has on the Nigerian nation.

*Source: NCCE (2012), p. 122–129; www.ncce.edu.ng.*

**TABLE 2:** Student learning outcomes for social studies (SLO).

SLO codes	Student learning outcomes
SLO 1	Demonstrate awareness and appreciation of the nature of Social Studies
SLO 2	Explain the basic concepts of man in the social environment
SLO 3	Apply the knowledge obtained in carrying out their daily activities and develop the right attitudes toward issue of environmental control and management
SLO 4	Develop scheme of work and lesson plan using NERDC curriculum as guide
SLO 5	Appreciate and demonstrate the need for national unity and integration in Nigeria
SOS 6	Appreciate the uniqueness inter-dependence and Universality of man
SOS 7	Able to know the dynamics of economic activities and demonstrate how they can contribute their quota to a stable economy
SLO 8	Comprehend the relevance of government in the society and the need to participate.
SLO 9	Demonstrate their awareness of the rule of law and how it relates to political issues.
SLO 10	Demonstrate methods and techniques necessary for the effective teaching and learning of Social Studies for Basic 7–9 Social Studies
SLO 11	Exposing students to principles of research and statistical methods for effective research work in Social Studies
SLO 12	Write a study-report on undertaking field exercise and develop learner’s skills for data collection
SLO 13	Appraise and address problems of National Development
SLO 14	Demonstrate positive qualities of good Citizenship
SOS 15	Appraise the structure, functions and problems of providing social services in Nigeria
SOS 16	Have and insight into who is responsible for promulgating and executing the laws of the society
SOS 17	Appraise the problems and prospects of transport and communication
SLO 18	Demonstrate positive attitudes toward family life.
SLO 19	Appraise the role of Nigeria in the international community
SOS 20	Proffer possible solutions to the problems affecting social institutions in Nigeria
SOS 21	Develop awareness and appreciation of the changes globalization has be on the Nigeria society.

*Source: NCCE (2012), p. 122–129; www.ncce.edu.ng.*

### Methods of Teaching and Learning Nigeria Certificate in Education Social Studies in Nigeria

The methods described here encourage pupils to become more involved with the ideas and information presented – much more involved than merely memorizing and reciting facts, dates, locations, products, etc. They allow learners to discuss, question and think about the things they are learning. One of the main purposes of Social Studies is to teach learners to think critically about the life of their community and the nature of their society. These methods help to achieve this goal by making them look closely at things that are around them – to study things with which they are somewhat familiar. By doing this learners can develop their reasoning and analytical powers, and then they can be helped to apply the same analytical abilities to things with which they are not at all familiar. In this way learners move from the known to the unknown with increased continuity and confidence (Iloeje and Okoro, 1977). These methods are not the only ones to be used in teaching Social Studies.

• Inquiry method is a method of conducting quests, searches into problems, investigate and studying the alternative solutions to any problems. The learners acquire the subject-matter by probing (Nwosu and Corbin, 1977).
• Small group discussion is a type of activity which involves breaking the class into small groups for effective talking about a topic, an issue, a problem, or a question. The size of the groups may vary, but they should have a group leader and a recorder of the discussion.
• Whole class discussion is the discussion carried out on whenever an entire class gathers as one unit. The teacher or a learner-leader sits within the circle or square to create an air or informality and help the group to take more responsibility.
• Sorting is a particular strategy, using picture, cards, photographs, clipping or other objects, in which the learners are required to arrange these things according to their common relationships, such as color, kind, class, group, use, origin, etc.
• Role-playing is a spontaneous dramatization of a situation to show emotional reactions and imagined behaviors. Learners may play the role of a parent or some other person and try to feel and behave as that person would do in that situation.
• Simulation game is a game played to enable learners to carry out real life situations. Through these games, learners pretend to represent or reproduce actual economic or political situations, important events, ideas, institutions, etc., the game is usually organized by both the teacher and the learners. Many different life situations could be represented in the form of simulation games.
• Resource person is a member of the community used by the teacher as a means of enlightening the learners on any leaning experiences. Sometimes the teacher can prepare a learner in the school to play the role of a resource person.
• A skit or playlet is a written play about some aspect of the subject matter presented as an event, situation, issue or problem. It is usually planned and rehearsed before being presented to the class. In a skit or playlet roles are described and actions are written on the script. The actors may memorize and recite the script, or read from it, as they perform the actions given in the script.
• Field trips, study trips or excursions is any learning activity that is carried on by the learners, as a group, outside the classroom, under the guidance of the teacher. Trips can be made to farms, factories, airports, health centers, police stations, etc.
• Project method is any method which engages learners in a task-centered learning activity, ideally one having a concrete result or end product. A project is intended to help the learner gain a more concrete understanding of an abstract or comprehensive idea. It should make the topic more relevant to real things and events that happen in everyday life (Aina et al., 1982).
• Problem-Solving method involves the use of a scientific approach to learning and teaching. The method enables the learner to become aware of the fact that there is an orderly procedure in thinking and doing things. Problem-solving should be student-oriented, and learner-centered (DuBey and Barth, 1980).
• Lecture method, sometimes referred to as the expository method, is characterized by the active teacher who does all the talking throughout the lesson and the passive learners who merely listen or take down notes of the important points in the lecture. In classes where the ideal lecture method is in use, it is not uncommon to find the class dull and drab, the lesson uninteresting and the students looking blank, empty, suspended and gloomy (DuBey et al., 1980). Lecture method has the advantage of being able to cope with large classes and faster teaching through the syllabus, though, of course, at the expense of much depth. This advantage is thus nothing compared with the resultant loss in quality and standard.

### Evaluation Approach to the Teaching and Learning Nigeria Certificate in Education Social Studies Education

The process of education includes three major divisions – formulation of objectives, designing learning experiences for the objectives, and assessing the outcomes of education. Evaluation is an important part of the whole program of education. There exists an inalienable three-fold relationship among objective (ends), teaching procedures or learning experiences (means) and evaluation (evidence). Evaluation is a process of determining how far the NCE Social Studies curriculum objectives have been attained. It means the finding out the strengths and weaknesses of the learners and total curriculum endeavor.

Evaluation is an inclusive concept – it indicates all kinds of efforts and means to ascertain the quality, value and effectiveness of desired outcomes. Evaluation in Social Studies involves identification and formulation of objectives of teaching Social Studies; their definition in terms of learner behavior, that is, what changes do we expect in the learner by each one of those objectives; and construction of valid, reliable and practical instruments for observing the specific phases of learner behavior such as knowledge, information, skills, attitudes, appreciations, personal-social adaptability, interests and work habits (Kochhar, 2001). An effective program of evaluation in Social Studies should include a wide range of devices – from observation to test items on skills and understandings, from role-playing to conferences, from diaries and personal inventories to different types of tests – essay, short-answer and objective. Only by such a comprehensive program can the wide range of goals be probed.

Evaluation strategies for measuring and evaluating learning outcomes in NCE Social Studies Education in Nigeria include:

a. Cognitive domain – easy type questions, objective type questions, assignment, class discussion (oral questioning), projects, etc.;
b. Psychomotor/skills domain – essay type questions, observation, class discussion, work samples, etc.; and
c. Affective domain – observation, rating scales, checklists, record of behavioral/anecdotal record, conferences or interviews, etc.

### Course Content and Description of Nigeria Certificate in Education Social Studies From the Minimum Standards

SOS 111 Foundations of Social Studies (2 Credits) Compulsory

This course is intended to expose students to the philosophy and basic characteristics of Social Studies education. At the end of the course students are expected to demonstrate awareness and appreciation of the nature of Social Studies:

- The definition and scope of Social Studies.
- The philosophical background of Social Studies.
- (a) In relation to the National Policy on Education.

(b) In relation to theory of Inter-relationships in learning.

- The concept of integration in Social Studies.
- The relationship between Social Studies, the Social Sciences, and other subjects.
- Aims and objectives of Social Studies.
- The relationship between Social Studies and Population, family Life, Drug, and AIDS Education.

SOS 112 Man and His Social Environment (2 Credits) Compulsory

This idea of man as a social being and why he lives as group is the focus of this course. At the end of the course, students are expected to:

- Explain the basic concepts of man in the social environment.
- Definition and types of man’s social environment.
- Why man lives in groups.
- Family types, structure, functions and changing roles.
- Forms and problems of marriage: customary, religious, and ordinance.
- Safe age for marriage, family formation, child bearing, and rearing practices.
- Primary and Secondary groups – definitions, characteristics, and functions.
- Kinship systems in Africa.
- Factors that promote living together: love, customs, morality, folkways, mores, and laws.
- Women education Family welfare.
- Gender roles.

SOS 113 Man and His Physical Environment (1 Credits) Elective

The course is designed to uplift the knowledge of students on the physical environment, how it influences and how man through his numerous activities influences the physical environment. As such, students are expected at the end of the course to:

a. Apply the knowledge obtained in carrying out their daily activities.
b. Develop the right attitudes toward issue of environmental control and management.

- The concept of physical environment: Minerals and Rocks; Relief features, soils; atmosphere, weather and climate; vegetation; water bodies (ponds, streams, rivers, lakes, lagoons, seas, and oceans). The influence of physical environment on man’s activities and vice-versa.

SOS 121 Introduction to the NERDC National Curriculum For Social Studies (2 Credits) Compulsory

The course introduces students to the NERDC National Curriculum of Social Studies for basic education 7 – 9. At the end of the course students should demonstrate their ability to develop a scheme of work and lesson plan based on the NERDC curriculum guide.

- An overview of NERDC Social Studies National Curriculum for Basic 7 – 9. Distinctions among curriculum, syllabus, scheme of work, unit plan and lesson plan; locating social studies syllabuses; preparation of lesson plans in Social Studies; distinction among teaching methods, techniques and strategies; an overview of Social Studies teaching methods; an overview of instructional resources in Social Studies; evaluation strategies in Social Studies, Micro-teaching (meaning and approaches).

*Note: The focus of this course should be on NERDC National Curriculum for Social Studies for Basic 7–9. Students should develop scheme of work and lesson plan using NERDC curriculum as guide.*

SOS 122 Nigeria as a Nation (1 Credit) Elective

The students are taken through the evolution of the Nigerian National and to appraise the cultural Diversities of our nation. At the end of the course, students should be able to appreciate and demonstrate the need for national unity and integration in Nigeria.

- The concept of nation.
- Nigeria as a geo-political entity.
- Ethnic groups in Nigeria (number, characteristics, and location).
- Population of Nigeria: size and distribution.
- Integration: Concept and forms.
- Efforts at national integration (national symbols, new capital city, constitutions, NYSC, Unity Schools, Federal Highways, etc.).
- Problems of national integration.

SOS 123 The Origin and Nature of Man (1 Credit) Elective

This course of designed to exposed students to the origin and nature of man. At the end of the course they are expected to appreciate the uniqueness inter – dependence and university of man.

- The various explanations of the origin of man namely; religious, mythical and scientific.
- The beginning of man from Apes to homo-sapiens.
- Harmonizing Forces (tool making, Language, Social Organization and Management of Man’s Prolonged Childhood).
- The uniqueness of man.
- The interdependence of man.
- Race and Racism.
- Humanity Universality.

SOS 124 Man and His Economic Activities (2 Credits) Elective

The focus of this course is to introduce the learners to the major economic activities within the Nigerian state. At the end of the course, the learners should be able know the dynamics of economic activities and to demonstrate how they can contribute their quota to a stable economy:

- Man’s basic economic problems; Scarcity, and choice.
- Factors of production.
- Man’s reactions to supply and demand of goods and services.
- Production systems: primary, secondary, and tertiary.
- Sources of government revenue in Nigeria.
- Economic problems: Inflation, unemployment, poverty and poverty alleviation programs.

SOS 125 Man and His Government (2 Credits) Compulsory

This course intends to expose the learners to the rudiments of governance in human society. At the end of the course, the learners should be able to comprehend the relevance of government in the society and the need to participate.

- The concepts and role of government in society.
- Power and Authority.
- Traditional forms of government: family, clan, village, town empire, etc.
- Modern forms of government - democracy, autocracy, monarchy, and the military.
- Organs of government - executive, legislative, judiciary, and the press.
- Tiers of government in Nigeria - Local, State and Federal emphasizing their structure and functions.

SOS 211 Nigerian Political Life (2 Credits) Compulsory

This course aims at exposing students to the concepts of the Nigerian political life in relation to the general provisions of the Nigerian Constitution. At the end of the course students are expected to demonstrate their awareness of the rule of law and how it relates to political issues.

- Nigerian Political Life.
- The concepts of nation, state and country.
- Nationalist movements and political parties before independence.
- Independence, the Republics and the political parties.
- Military Rule in Nigeria.
- Political Issues (Population size, power sharing/shift, revenue allocation, resource control, etc.).
- Constitutions (meaning, purposes and types).
- Constitutional developments in Nigeria since 1914.
- General provision of the current Nigerian constitution (Fundamental objectives and directive principles of state policy, citizenship, fundamental human rights, Arms of Government, FCT, and General supplementary provision.)

SOS 212 Practicum for National Curriculum For Basic 7–9 (2 credits) Compulsory

This course aims at exposing students to Practical application of NERDC National Curriculum for Social Studies. At the end of the course students should be able to demonstrate methods and techniques necessary for the effective teaching and learning of social studies for basic 7–9. Social Studies.

- Methods and techniques necessary for the effective teaching of Social Studies for Basic 7–9. Dramatic representation, discussion, creative activities, simulation, problem solving, questioning, technique, concept mapping, etc.

Emphasis should be more on practical than theory.

*Note: The mode of assessment for this course should be practical application of NERDC curriculum for Social Studies Basic 7–9 to develop:*

• Scheme of Work (In group).
• Lesson plan (Individual).
• Micro-Teaching (Presentation of two topics).

SOS 213 Social Studies Research Methods and Statistics (2 Credits) Compulsory

This course aims and at exposing students to principles of research and statistical methods for effective research work in social studies.

A. Research

- Concept and content of research:

Types of research
Choice of research topic
Purposes/objectives of research

- Review of relevant literature.
- Research methodology (Research Design):

Stating research problem
Choice of population
Sample and sampling techniques
Hypothesizing
Data collection techniques:
Observation, interview, questionnaire, etc.
Organization and presentation of data and statistical representation.
B. Appendices

- Bibliography and

References

Statistic: Meaning, Types and Uses

- Descriptive statistics:
  Measures of central tendency
  Measures of variability

- Inferential statistics:
  Parametric and non-parametric

SOS 214 Field Trip (2 Credits) Compulsory

- The course will afford the students the opportunity to visit both far and near environment in terms of educative interest in Social Studies. Students will be out for 1 to 4 days of studying both physical and social phenomenon, human activities in terms of housing, occupational practices, dressing, culture, etc. Students will be able to write a study-report on undertaking field exercise. And by so doing develop in learners skills of data collection, e.g., interceding, documentation and reporting.

SOS 221 Issues and Problems of National Development and Modernization (2 Credits) Compulsory

The course is designed to expose students to basic concepts of National Development. At the end of the course, students will be able to appraise and problems of National Development.

- Nature and concepts of national development.
- Meaning, nature and relationship between modernization and national development.
- Dimensions of national development (economic development, political development, social development, legal development, educational development, technology and health, etc.).
- Problems of national development (poor data base, corruption, poor plan implementation, external manipulations and illiteracy, etc.).
- Factors and processes of modernization.
- Aspects of modernization (population, urbanization, education, science and technology, socio-cultural political and economic).

SOS 222 Citizenship Education (2 Credits) Compulsory

The course introduces students to some concepts of citizenship education. By the end of the course, students will demonstrate positive qualities of good citizenship.

- The concept of socialization.
- Types of socialization (Primary, secondary, and adult).
- Agents of socialization (Family, peer group, school, mass media, church, mosque, etc.).
- Processes of socialization.
- Political socialization and mass mobilization (MAMSER, NOA, etc.).
- Problems of socialization.
- The role of Social Studies in the socialization and production of good citizens.
- The concepts of citizen and citizenship education.
- Types of citizenship (single and dual).
- Citizenship acquisition in Nigeria (By birth, by registration and by national naturalization).
- Renunciation and denial of citizenship.
- Qualities and duties of a good citizen.
- Fundamental Human Rights.
- Lawful denial of fundamental human rights.
- Violation and protection of Human Rights.
- Ways in which human rights are violated.
- Ways of protecting Human Rights.

SOS 223 Social Services in Nigeria and Social Change in Nigeria (1 credit) Elective

This course focuses on the institutions that provide public utilities and the factors and processes of social change. At the end of the course the learner should be able to appraise the structure, functions and problems of providing social services in Nigeria. Similarly, the students should be able to make critical examination of the factors and processes of social change in Nigeria.

- Social administration and social policies defined.
- Educational institutions: Structures and functions in Nigeria.
- Health institutions: Structure and functions, National AIDS/STD Control Programs in Nigeria (NASCP).
- Housing Policy
- Other services and utilities: Fire, Prison, Postal, Old age pension, Nigeria Police Force, Water Supply, Electricity, Transport, Communication.
- Attitude to public utilities.
- Population pressure on social services in Nigeria.
- The concept of change.
- Theories of change.
- Types of change.
- Factors and processes of change.
- Changes in Nigeria before and after 1960 in demographic, economic, socio-cultural and political system.
- Change and its effects on the individual and the family in Nigeria.

SOS 224 Law Related Education (1 Credit) Elective

- The course seeks to describe the element of laws, rules, regulations ordinances, edicts, decrees, norms and moves as it affect the modern society.
- This course also looks at the sources of the Nigeria law for the purpose of making the learner have an insight into who is responsible for promulgating and executing the laws of the society.
- This is done by introducing the learner to the constitutions of the Nigerian government, colonial heritage, traditions and sharing.
  • the process of law making in Nigeria;
  • litigations, criminal and civil cases; and
  • Administration of justice; the function of the police, courts and law and prisons services.
- The course will equally take a critical look at the role of the judiciary in the implementation of the law, e.g., the hierarchy of courts, personal and independence.
- A detail study of “You and the Law.”

SOS 225 Transport and Communication (1 Credit) Elective

The course seeks to expose students to various means of transportation and communication. At the end of the course, students should be able to appraise the problems and prospects of transport and communication.

- The differences between transportation and communication.
- Traditional and modern means of Transportation: Advantages and problems.
- Traditional and Modern means of Communication: Advantages and problems (E-mail, fax, telex, radio, and internet). Practical application should be demonstrated to students.
- The role of transportation and communication on national development.
- The mass media-what is mass media, their role in national development. Problems, etc.
- Students should develop case studies materials on any mass media of their choice (It should form part of student C.A).

SOS 321 Population and Family Life Education (2 Credits) Compulsory

The course focuses attention on population and family life education. At the end of the course, students should be able to demonstrate positive attitudes toward family life.

- The concept of population.
- The concept of family life.
- The family life education.
- The objective of population education.
- The objective of family life education.
- Gender issues and family life education.
- Family size and welfare.
- The roles of members of the family.
- The responsibility of parenthood.
- Population data, i.e., census and vital registration.
- Population distribution in Nigeria and Africa.
- The relationship between Social Studies and Population, Family Life, and Aids Education.
- National Population Policy (NPP).
- Population dynamics: growth, decline and structure and their socio-economic implication.
- Methods of teaching Population/Family life Education.

SOS 322 Nigeria External Relations (2 Credits) Compulsory

The course focuses on the principles of International relations and Nigeria’s foreign policy. At the end of the course, students should be able to appraise the role of Nigeria in the international community.

- The concept of Internal Relation.
- Nigerian foreign policies (Principles and Policies).
- Nigeria and ECOWAS (Formation, functioning, and problems).
- Man in International Community.
- World Tension: Causes and solutions (games, conferences and membership, etc.).
- Nigeria in the Common Wealth.
- Nigeria in OPEC.
- Nigeria in the UNO (contribution) benefit and problems.
- Nigeria in Africa Union.

SOS 323 Social Institutions (1 Credit) Elective

The course is designed to expose students to be structure, functions and problems of different social institutions in Nigeria. At the end of the course, students should be able to proffer possible solutions to the problems affecting social institutions in Nigeria.

- The concept of social institution.
- Structure and functions of different social institutions such as legal political, economic, religious, educational, health institutions, etc., in Nigeria.
- Problems of social institutions in Nigeria.
- Religion in Society.
- Religion in Nigeria.
- Religion and Morality.
- Religion and Politics.
- Conflict and tolerance in Nigeria.

SOS 324 Globalization (1 Credit) Elective

This course aims at exposing students to the concepts of globalization in relation to the impact it has on the Nigerian nation. At the end of the course, students are expected to develop awareness and appreciation of the changes globalization has be on the Nigerian society.

- The concept of Globalization.
- Historical antecedents (colonialism, Imperialism, Europeanization, Westernization, Americanization, etc.).
- Who is globalizing, and what is being globalized?
- Who is globalizing, and what cannot be globalized?
- Impact of globalization on the South (i.e., Developing and Underdeveloped countries, including Nigeria).
- What can Nigeria globalize? How? (i.e., Nigeria and the globalization process).
- *The objectives of NCE Social Studies education from the Minimum Standards.*
- *Developing curriculum coverage map.*

When creating a curriculum map, every subject in program and program attributes are correlated to control association (Joyner Melito, 2016b). The data is given in a map so that the inclusion of the whole program, gaps and redundancies can be watched in one copy. Social Studies Department, FCT COE Zuba, Abuja has a 3-year history of curriculum mapping, with a reputation for academic excellence in Nigeria, has 2,156 students enrolled in the 3-year program, 11 qualified teachers, 06 have a master’s degree and 05 have a doctoral degree preceding to the present investigation. Purposeful sampling approach was adopted in selecting the location of investigation (Shilling, 2013). Jacobs (1997) indicates that mapping needs to be customized to specific schools, including specific grades or teams, so teachers need to decide what’s important to them in order for the participants to have control of the process.

Porter (2002) explains three major instruments of assessing program topic and association between topic and attributes: (1) studies for teachers on topics of the subject, (2) studies of attributes including evaluations and (3) instruments of measuring association among subjects and learning. The aim of these instruments is to explain subject in a global understanding so that association can be decently measured (Porter, 2002). While these instruments do take significant time to develop and refine, the data they generate provides a wealth of knowledge on the current practices in the curriculum and are invaluable for assessing outcome alignment (Joyner Melito, 2016a).

Collection of every subject in the program and program attributes are examined to canvas for association during the generation of program map. This information can be presented in a grid so that the coverage of the entire curriculum can be viewed in a single image (Joyner Melito, 2016a). Challenges with learners can come up when learners are required to have a difficult grade of ability to enter a subject more than their real ability grade. Nevertheless, if learners have a difficult attribute grade that is required from them, they will be tired and discouraged in the course (Ambrose et al. in Joyner Melito, 2016a). Thence, it is useful to comprehend learner ability levels, understand student competency levels and their fluctuations at all levels as they develop across the program so that text books can be adequately channeled to meet the needs of the learners based on grade of attribute. Attributes for NCE Social Studies Minimum Standards were generally written to include all Social Studies content. To enable a more detailed look at the current NCE Minimum Standards, Social Studies Core Competencies and electives (Table 3) were used in mapping practice instead of the minimum standards attributes (Joyner Melito, 2016a). Using Social Studies Core Competencies and electives helped review program to verify compliance with benchmarks to ensure audit.

**TABLE 3:** Course titles, social studies (SOS) core competency category and electives.

Course codes	Course titles
SOS 111	Foundation of Social Studies
SOS 112	Man and His Social Environment
SOS 113	Man and His Physical Environment
SOS 121	Introduction to the NERDC National Curriculum for Social Studies
SOS 122	Nigeria as a Nation
SOS 123	The Origin and Nature of Man
SOS 124	Man and His Economic Activities
SOS 125	Man and His Government
SOS 211	Nigerian Political Life
SOS 212	Practicum for National Curriculum for Basic 7–9
SOS 213	Social Studies Research Method and Statistics
SOS 214	Field Trip
SOS 221	Issues and Problems of National Development and Modernization
SOS 222	Citizenship Education
SOS 223	Social Services in Nigeria and Social Change in Nigeria
SOS 224	Law Related Education
SOS 225	Transport and Communication
SOS 321	Population and Family Life Education
SOS 322	Nigeria External Relations
SOS 323	Social Institutions
SOS 324	Globalization

*Source: NCCE (2012), p. 122–129; www.ncce.edu.ng.*

In creating the program maps, Lecturers presented NCE Minimum Standards of Social Studies course(s) to the teacher controlling mapping practice. The teacher controlling mapping practice went through the Minimum Standards and generated tentative cover map of Social Studies compulsory attributes and electives for every course. The courses were divided into categories according to Social Studies compulsory attributes and electives to ascertain the map. Each category was verified differently. Course lecturers in every category came together with head of mapping concurrently consider which Social Studies compulsory attributes and electives were included in the course they teach for every category. Lecturers that were absent from the meeting were personally asked to ascertained the cover of compulsory attributes and electives utilized in their NCE Social Studies course (s).

The Department of Social Studies’ Minimum Standards is developed to offer learners composite cover of Social Studies’ insight, prospects to use facts learning in actual-global situations, promote acquisition of knowledge, attitudes, values, appreciations and skills necessary for developing social and civic responsibilities (NCCE, 2012)^1^. Preferably, the whole Social Studies courses should be associated with Department of Social Studies’ Minimum Standards attributes. Secondly, complete Minimum Standards should include Social Studies compulsory attributes and electives for learners to proof proficiency in ability by the completion of the program. The full covered mapping for all NCE Social Studies Minimum Standards is shown in Supplementary Table 1.

### Research Methodology

This study used the documentary research method. In this research, the term document is used to refer to official written texts available for public use and are secondary sources of data intended for specific audiences (Joskin, 2013). Atkinson and Coffey in Joskin (2013) argued that records are considered social facts created to be used for social contexts in an organized manner and can be subjects of research making them subject to interpretations. In this study two documents define the policy curriculum. These are The Nigeria Certificate in Education Minimum Standards (NCCE, 2012) and The Social Studies Curricula (NCCE, 2012). The two policy documents provided the backdrop to teach and learn Social Studies in FCT College of Education Zuba. Burns in Joskin (2013) opined that documents such as policies are considered more credible than other documents because of their authority. Cohen, et al in Joskin (2013) stress that documents are socially created products of social institutions to serve their purpose and that there may be bias elements encrypted in documents that may not serve the purposes of research well. Alternatively, Scott in Joskin (2013, p. 116) suggests four criteria for assessing the quality of documents and these were embraced by this study:

• Authenticity – is the document legitimate and original?
• Credibility – is the document error free and has no distortion?
• Representativeness – is the document representing accuracy the position of the writer (s)?
• Meaning – is there clarity and comprehension seen in the evidence?

Qualitative documents enable a researcher to obtain the participants’ language and words, an unobtrusive source of information; represents data that is thoughtful in those participants have taken care to compile them; and as written evidence, it serves the time and expense of transcribing it to a researcher (Creswell, 2009).

The purposeful sampling strategy was used to select the research site (Shilling, 2013). The NCE program is 3 years and the site was Department of Social Studies, Federal Capital Territory (FCT) College of Education Zuba, Abuja, Nigeria. The department has 3-year history of curriculum mapping. The school is located in Federal Capital Territory of Nigeria and has a reputation for academic excellence in the Territory and across the Nation. Jacobs (2004) suggests that mapping needs to be tailored to individual schools, even to individual grades or teams because teachers must determine what is important to them in order that ownership of the process belongs to the participants. The course curricula of Colleges of Education in Nigeria are comprehensive documents detailing the knowledge, skills and competences, as well as appropriate attitudes which students are expected to acquire by pursuing a given program of study. The Minimum Standards document was used as benchmark by NCCE for moderating program in Colleges of Education in Nigeria.

Quantitative information was analyzed applying descriptive frequencies and percentages, as curriculum mapping is not usually a process studied with parametric statistics (Hubball and Burt, 2007; Ramia et al., 2016). Potential gaps were defined as coverage for each competency in about ≤20% of the courses and potential redundancies was considered as coverage of ≥80% of the courses (Joyner Melito, 2016a).

### Gap/Redundancy Map

Jacobs (2004) has asserted curriculum mapping as a tool and a mechanism that can be integrated into the school to assist in the coordination of the three primary components of successful student learning: the expected (curriculum) for the provided (instruction) and the achieved (assessment). Liu et al. (2010) argued that curriculum mapping procedure assists teachers detect alignment, gaps, duplications, discrepancies and benefits with programs when done in a collaborative format.

Hale in Joyner Melito (2016a) defines a gap as knowledge or skills necessary for the complete understanding of materials or the complete creation of skills that are not taught or not taught with the scope needed while overlap is a layoff attributes skills in more than two courses. Whereas few reiterations are required for lessons, data retrieval, strengthening foundation for current insight, this duplication is not inherently overlap (Joyner Melito, 2016a). Hale in Joyner Melito (2016a) agreed that redundancy does not promote improved competencies or further learning. Deficiency/overlap was analyzed on Social Studies curricula during the curriculum mapping process to determine whether the competencies received excessive coverage or lacked coverage. In addition, it investigated possible holes and redundancies to assess whether the gap or redundancy actually existed because NCCE (2012) (see text footnote 1) posit that “various methodological approaches should be adopted in teaching NCE Social Studies with special emphasis on *inquiry* and *field trip*” (p. 110). Curriculum map for gap/redundancy analysis is shown in Supplementary Table 1.

## Outcomes and Assessment Results

a. The coverage map showed that the whole attributes were included at a minimum of one course.
b. The course curricula show obvious gaps in the scope of competencies.
c. There is no alignment between the learning outcomes of the student and the content of the course.
d. Social Studies Research Methods and Statistics are not enshrined in other courses in Social Studies Department.
e. Field trip is not enshrined in other courses in Social Studies Department.
f. Cooperation and collegiality among the faculty is low.
g. There is no substantial coherence between the student learning outcomes and the curricula of the course.
h. The College Management has no political will to sponsor the faculty for curriculum mapping, field trips, workshops and seminars.
i. The faculty has insufficient curriculum mapping professional development.
j. Virtual education is not fully implemented in the College.

## Discussion

Curriculum mapping shows teachers how their instruction fits into the overall curriculum picture and how they discuss their subject in other curriculum areas (Romkey and Bradbury, 2007). Acknowledging that curriculum mapping as a process and tool provides a holistic and comprehensive view of the curriculum across all the subject areas and levels of study (Madiba, 2011) and that it enables academic staff to address key pedagogical issues by fostering debate and reflection, the researchers used curriculum maps as a means of visual communication. Curriculum maps, consisting of the key aspect such as learning outcomes, the knowledge and skills to be acquired, teaching events, learning activities and assessment tasks, allow academics to obtain a holistic view of the curriculum.

Curriculum mapping strengthens the curriculum and plan by defining the differences, overlaps, consistencies and strengths of a suggested intervention (Lam and Tsui, 2013). Curriculum mapping is the process of indexing or diagramming a curriculum to identify and address academic gaps, redundancies, and misalignments for purposes of improving the overall coherence of a course of study and, by extension, its effectiveness (a curriculum, in the sense that the term is typically used by educators, encompasses everything that teachers teach to students in a school or course, including the instructional materials and techniques they use). Banta and Blaich (2010) refer to the closure of the loop as the process of evaluating results, identifying areas for improvement, and implementing these improvements. In order to be effective in mapping procedures, this process ought to be developed and sustained for steady program development.

One of the major benefits of curriculum mapping is aligning curriculum with state standards and assessment practices. In the educational literature, curriculum alignment is described as a process that guides teaching and learning by ensuring agreement between curriculum, state standards, classroom practices, and assessment (English, 2000). Evidence of this alignment should be present in both the written and taught curriculum (Jacobs, 2004). Vertical alignment helps teachers make sure there are no gaps, repetitions, or redundancies at different grade levels and there is smooth and sensible spiraling of curriculum (Udelhofen, 2005). Curriculum that is aligned with standards and it is taught with fidelity to standards will help students achieve their learning objectives (English, 2000). Curriculum mapping is an active process for aligning learning outcomes and curriculum activities among students (Harden, 2001). Curriculum mapping aims to ensure that teaching is purposefully structured and logically sequenced across grade levels so that students are building on what they have previous learned and learning the knowledge and skills that will progressively prepare them for more challenging, higher-level work. Research indicates alignment is a powerful indicator of academic achievement.

When a curriculum is coherent within a subject area; it may be aligned both within and across grade levels. Curriculum mapping for subject-area coherence aims to ensure that teachers are working toward the same learning standards in similar courses, and that students are also learning the same amount of content, and receiving the same quality of instruction, across subject-area courses. Curriculum mapping for interdisciplinary coherence may focus on skills and work habits that students need to succeed in any academic course of discipline, such as reading skills, writing skills, technology skills, and critical-thinking skills.

Field trips are learning activities that are carried on by learners, as a group, outside the classroom, under the guidance of the teacher because Social Studies is the study of human beings and their environment. In the process, learners gain first hand information about some concepts, and have opportunity to extent their knowledge of their environment (Nzeribe, 2002). For example, learners may have some misunderstanding about what doctors and pharmacists do but with a visit to the hospitals, such learners have the opportunity to learn firsthand about the duties of doctors and pharmacists. It offers an opportunity for learners to observe directly and have a personal experience of places which they visit. It makes for correlation of school subjects (Nwosu and Corbin, 1977).

A curriculum map is useful in acting as a successful venue for fostering conversation about curriculum and instruction among all faculty members. Gross (2001) also pointed out that apart from being an effective means toward achieving alignment among curriculum, instruction, and assessment, curriculum mapping facilitates professional dialog that empowers both teachers and administrators and help them create a cohesive educational program for learners. Curriculum mapping is a vehicle through which teachers communicated curriculum and instruction. It is very important to create collaborative teams when the curriculum mapping process unfolds within a school. Curriculum mapping as a process, as defined by Madiba (2011: 381) enables academic staff to have rich and authentic conversations’ about their existing curriculum orientations and teaching practice. He further describes curriculum as a reliable mechanism to steer the teaching and learning agenda toward achieving the desired outcomes.

Learning outcomes can be embedded as nodes in a curriculum map (Harden, 2001). This can be used to plan progress through the curriculum and to relate learning experiences to the outcomes as developed in the Department of Social Studies. This progression is consistent with the concept of a spiral curriculum which is characterized by the revisiting of topics at an increasing level of difficulty, the relation of new learning to previous learning and an increasing level of competence in students (Harden and Stamper, 1999). Learning outcomes embedded in a map provide students with an understanding of where they are going and the steps that they need to make to progress to their destination. In this way the student can judge the relevance or otherwise of the various learning opportunities presented to them. The goal of mapping each individual activity and identifying where learning outcomes are achieved is to stress the activity’s learning goals (Manogue and Brown, 2007). Thus, the learning outcomes that students achieve at the close of their program of study offer the context within which curricula are developed, given, ensured, and finally evaluated (Shumway and Harden, 2003).

Leadership must be ongoing for curriculum mapping initiative to succeed and sustain. The leadership structure should make sure they provide enough assistance and support, maintain constant communication, and monitor the progress of the initiative. Teacher commitment and motivation for the curriculum mapping initiative increase if there is follow-up and monitoring from the leaders and administrative staff (Huberman and Miles, 1984). Many methods of curriculum mapping are largely based on the teacher’s experience, except for the experience gained through face-to-face deliberation (dialog and discussion) (Lam and Tsui, 2013). Taking into account that the main aim of curriculum change at our institutions is to empower academic staff to become reflective professionals, curriculum mapping is used both as a process and an evaluation tool to encourage academics to change their curriculum orientation from a product-centered to a process-centered approach.

Curriculum mapping enables all participants to examine or re-examine their individual and collective beliefs regarding teaching and learning in a structured and supportive environment (Uchiyama and Radin, 2009). Hale (2008) says this, “curriculum mapping is not a spectator sport. It demands teachers’ ongoing preparation and active participation. There must also be continual support from administrators who have a clear understanding and insight into the intricacies of the mapping process.”(p. 15). Curriculum mapping is a scheme of work developed by teachers to explain the subject topics and planned learning outcomes set for the school’s semester or academic year, the main points of teaching, their respective teaching activities and tools, and the assessment methods for various topics (Lam and Tsui, 2013).

Changes in the main course content may necessitate revisions to the virtual education course study guide and learning outcomes mapping. This may now be managed as part of our organized rolling review process, which can be improved further by the use of curriculum mapping tools (Kruger and Tennant, 2012; Walton, 2014). Jacobs (2004) states, “…curriculum maps have the potential to become the hub for schools into a central database that can be accessed from anywhere through the internet can provide relief …Mapping becomes an integrating force to discuss not only curriculum issues, but also programmatic ones.” (p. 126).

## Conclusion and Recommendations

The curriculum mapping and assessment activities described in this paper are an important part of ensuring curriculum learning outcomes are met, the courses in the curriculum combine into a cohesive whole, and changes to the curriculum are improving student learning. Because course content, course instructor, and general course offerings change over time, curriculum mapping is not simply a one-time exercise (Hale, 2008). Rather, the curriculum map should be a living document that is updated and assessed regularly (preferably annually) to ensure that the curriculum is continually in alignment with curriculum learning outcomes and no gaps or redundancies in competency coverage develop (Harden, 2001). Curriculum assessment also needs to be completed on a regular basis to monitor student progress through the curriculum, determine if curriculum competencies are being met, uncover trouble areas such as knowledge or skill gaps, and evaluate the effectiveness of changes made to the curriculum. The Department of Social Studies, FCT College of Education Zuba, Abuja will continue to update the curriculum map and assess learning outcomes on an annual basis to monitor student performance and determine if changes made are effective in promoting student learning.

We believe that virtual education course development can improve program accessibility, showcase external regulatory standards, and increase quality assurance without disrupting present material in well-designed and currently effective curricula. We can sustain the spirit of our program while prioritizing areas of greatest need and responding to external stakeholders without having to rewrite our entire curriculum by using virtual education courses. It is indeed useful and lowers the risk connected with creating a new course from scratch. Developing a virtual course provides an opportunity to raise the profile of a specific theme of NCE Social Studies graduate learning and teaching within a program, whilst maintaining the integrity of the current courses within the program.

Overall, curriculum mapping and assessment serves the purpose of monitoring student learning progress to ensure students gain the knowledge and skills required for success in their future career path.

Some recommendations below are made to remedy the coverage gaps and potential redundancies:

• NERDC National curriculum should be enshrined into the other pre-existing courses in the Department for adequate map coverage.
• Social Studies Research Methods and Statistics should be enshrined into other courses in Social Studies Department.
• Field trip should be enshrined in other courses in Social Studies Department.
• More assessment is also needed in each course to determine the emphasis of critical thinking.
• The faculty should be sponsored by the College management to attend workshops/seminar and field trips to upgrade their professional development on curriculum mapping.
• Curriculum mapping should be revisited and fully implemented in Social Studies Department. The lecturers should map the curriculum on semester basis.

We would like to recommend virtual education as a way for Program Directors to highlight elements of vertical and horizontal integration, whether the structure is modular or non-modular (Manogue et al., 2011; Oliver et al., 2011).

The researchers recommended further studies on the “Perception of teachers’ on the efficacy of curriculum mapping as a tool for planning and curriculum alignment in FCT College of Education Zuba, Abuja, Nigeria.”

## Data Availability Statement

The datasets presented in this study can be found in online repositories. The names of the repository/repositories and accession number(s) can be found in the article/Supplementary Material.

## Ethics Statement

The studies involving human participants were reviewed and approved by Near East University Ethical Committee Board. The patients/participants provided their written informed consent to participate in this study.

## Author Contributions

All authors wrote sections of the manuscript, manuscript revision, read, and approved the submitted version.

## Conflict of Interest

The authors declare that the research was conducted in the absence of any commercial or financial relationships that could be construed as a potential conflict of interest.

## Publisher’s Note

All claims expressed in this article are solely those of the authors and do not necessarily represent those of their affiliated organizations, or those of the publisher, the editors and the reviewers. Any product that may be evaluated in this article, or claim that may be made by its manufacturer, is not guaranteed or endorsed by the publisher.

## Footnotes

1
http://www.ncceonline.edu.ng/
