# Supplementary material for: Using Curriculum Mapping as a Tool to Match Student Learning Outcomes and Social Studies Curricula
Source: Front Psychol. 2022 Aug 18;13:850264. doi: 10.3389/fpsyg.2022.850264 (PMC9435443; doi:10.3389/fpsyg.2022.850264)
Supplement: Supplementary file 1 [file Table_1.DOCX]

**Supplementary Table - NCE Social Studies minimum standards basic coverage map and gap/redundancy analysis**

| **NCE Social Studies Course Codes** | | | | | | | | | | | | | | | | | | | | | | | |
| --- | --- | --- | --- | --- | --- | --- | --- | --- | --- | --- | --- | --- | --- | --- | --- | --- | --- | --- | --- | --- | --- | --- | --- |
| Student  Learning Outcomes (SLO) | Shortened competency | SOS 111 | SOS 112 | SOS 113 | SOS 121 | SOS 122 | SOS 123 | SOS 124 | SOS 125 | SOS 211 | SOS 212 | SOS 213 | SOS 214 | SOS 221 | SOS 222 | SOS 223 | SOS 224 | SOS 225 | SOS 321 | SOS 322 | SOS 323 | SOS 324 | % coverage  showing  Gap/redundancy  analysis |
| Demonstrate  awareness and appreciation  of nature  of Social Studies | Definition & scope of Social Studies. | X |  |  |  |  |  |  |  |  |  |  |  |  |  |  |  |  |  |  |  |  | **5%** |
|  | Philosophical background of Social Studies (SOS) in relation to the National Policy on Education. | X |  |  |  |  |  |  |  |  |  |  |  |  |  |  |  |  |  |  |  |  | **5%** |
|  | Philosophical background of SOS in relation to theory of Inter-relationships in learning. | X |  |  |  |  |  |  |  |  |  |  |  |  |  |  |  |  |  |  |  |  | **5%** |
|  | Concept of integration in Social Studies | X |  |  |  | X |  |  |  |  |  |  |  |  |  |  |  |  |  |  |  |  | **5%** |
|  | Relationship between Social Studies, the Social Sciences and other subjects | X |  |  |  |  |  |  |  |  |  |  |  |  |  |  |  |  |  |  |  |  | **5%** |
|  | Aims & objectives Social Studies | X |  |  |  |  |  |  |  |  |  |  |  |  |  |  |  |  |  |  |  |  | **5%** |
|  | Relationship between SOS & Population, Family Life, Drug and AIDS Education | X |  |  |  | X |  |  | X | X |  |  |  | X |  | X |  |  | X |  |  |  | 33% |
|  |  |  |  |  |  |  |  |  |  |  |  |  |  |  |  |  |  |  |  |  |  |  |  |
| Explain the basic concepts of man  in the social  environment | Definition & types of man’s social environment | X | X |  |  |  |  |  |  |  |  |  |  |  | X | X |  |  |  |  | X |  | 24% |
|  | Why man lives in groups |  | X |  |  | X |  |  |  |  |  |  |  |  | X |  |  |  |  |  |  |  | **14%** |
|  | Family-types, structure, functions & changing roles | X | X |  |  |  |  |  | X |  |  |  |  |  | X |  |  |  | X |  |  |  | 24% |
|  | Forms& problems of marriage: customary, religious & ordinance |  | X |  |  |  |  |  |  |  |  |  |  |  |  |  |  |  | X |  | X |  | **14%** |
|  | Safe age for marriage, family formation, child bearing & rearing practices | X | X |  |  |  |  |  | X |  |  |  |  |  |  | X |  |  | X |  |  |  | 24% |
|  | Primary & Secondary groups – definition, characteristics & functions |  | X |  |  | X |  |  |  |  |  |  |  |  | X |  |  |  |  |  |  |  | **14%** |
|  | Kinship system in Africa |  | X |  |  |  |  |  |  |  |  |  |  |  |  |  |  |  |  |  |  |  | **5%** |
|  | Factors that promote living together: Love, Customs, Morality, Folkways, Mores & Laws. |  | X |  |  |  |  |  |  |  |  |  |  |  |  |  | X |  |  |  | X |  | **14%** |
|  | Women Education & Family Welfare | X | X |  |  |  |  |  |  |  |  |  |  |  |  |  |  |  | X |  |  |  | **14%** |
|  | Gender roles |  | X |  |  |  |  |  |  |  |  |  |  |  |  |  |  |  | X |  |  |  | **10%** |
|  |  |  |  |  |  |  |  |  |  |  |  |  |  |  |  |  |  |  |  |  |  |  |  |
| Apply the knowledge and  develop the right attitudes towards issue of environmental  control and  management | The concept of physical environment: Minerals and rocks |  |  | X |  |  |  |  |  |  |  |  |  |  |  |  |  |  |  |  |  |  | **5%** |
|  | The concept of physical environment: Relief features |  |  | X |  |  |  |  |  |  |  |  |  |  |  |  |  |  |  |  |  |  | **5%** |
|  | The concept of physical environment: Soils |  |  | X |  |  |  |  |  |  |  |  |  |  |  |  |  |  |  |  |  |  | **5%** |
|  | The concept of physical environment: Atmosphere |  |  | X |  |  |  |  |  |  |  |  |  |  |  |  |  |  |  |  |  |  | **5%** |
|  | The concept of physical environment: Weather and climate |  |  | X |  |  |  |  |  |  |  |  |  |  |  |  |  |  |  |  |  |  | **5%** |
|  | The concept of physical environment: Vegetation |  |  | X |  |  |  |  |  |  |  |  |  |  |  |  |  |  |  |  |  |  | **5%** |
|  | The concept of physical environment: Water bodies (ponds, streams, rivers, lakes, lagoons, seas and oceans). |  |  | X |  |  |  |  |  |  |  |  |  |  |  |  |  |  |  |  |  |  | **5%** |
|  | The influence of physical environment on man's activities and vise - versa. |  |  | X |  |  |  |  |  |  |  |  |  |  |  |  |  |  |  |  |  |  | **5%** |
|  |  |  |  |  |  |  |  |  |  |  |  |  |  |  |  |  |  |  |  |  |  |  |  |
| Develop scheme of work and lesson plan  using NERDC curriculum as  guide | An overview of NERDC Social Studies National Curriculum for Basic 7 - 9. |  |  |  | X |  |  |  |  |  |  |  |  |  |  |  |  |  |  |  |  |  | **5%** |
|  | Distinctions among curriculum, syllabus, scheme of work, unit plan and lesson plan |  |  |  | X |  |  |  |  |  |  |  |  |  |  |  |  |  |  |  |  |  | **5%** |
|  | Locating Social Studies syllabuses |  |  |  | X |  |  |  |  |  |  |  |  |  |  |  |  |  |  |  |  |  | **5%** |
|  | Preparation of lesson plans in Social Studies |  |  |  | X |  |  |  |  |  |  |  |  |  |  |  |  |  |  |  |  |  | **5%** |
|  | Distinction among teaching methods, techniques and strategies |  |  |  | X |  |  |  |  |  | X |  |  |  |  |  |  |  | X | X |  |  | **19%** |
|  | An overview of Social Studies teaching methods |  |  |  | X |  |  |  |  |  | X |  |  |  |  |  |  |  |  | X |  |  | **14%** |
|  | An overview of instructional resources in Social Studies |  |  |  | X |  |  |  |  |  | X |  |  |  |  |  |  |  |  | X |  |  | **14%** |
|  | Evaluation strategies in Social Studies |  |  |  | X |  |  |  |  |  |  |  |  |  |  |  |  |  |  |  |  |  | **5%** |
|  | Micro-teaching (Meaning & Approaches) |  |  |  | X |  |  |  |  |  |  |  |  |  |  |  |  |  |  | X |  |  | **10%** |
|  |  |  |  |  |  |  |  |  |  |  |  |  |  |  |  |  |  |  |  |  |  |  |  |
| Appreciate and demonstrate the need for national  unity and  integration in  Nigeria | The concept of nation |  |  |  |  | X |  |  |  | X |  |  |  |  |  |  |  |  |  |  |  |  | **10%** |
|  | Nigeria as a geo-political entity |  |  |  |  | X |  |  |  |  |  |  |  |  |  |  |  |  |  |  |  |  | **5%** |
|  | Ethnic groups in Nigeria (number, characteristics and location) |  | X |  |  | X |  |  |  |  |  |  |  |  | X |  |  |  |  |  |  |  | **14%** |
|  | Population of Nigeria: Size and distribution | X |  |  |  | X |  |  |  | X |  |  |  | X |  | X |  |  | X |  |  |  | 29% |
|  | Integration: Concept and forms | X |  |  |  | X |  |  |  |  |  |  |  |  |  |  |  |  |  |  |  |  | **10%** |
|  | Efforts at national integration (national symbols, new capital city, constitutions, National Youth Service Corps (NYSC),Unity Schools, Federal Highways etc ) | X |  |  |  | X |  |  |  | X |  |  |  |  |  |  |  |  |  |  |  |  | **14%** |
|  | Problems of national integration. | X |  |  |  | X |  |  |  |  |  |  |  |  |  |  |  |  |  |  |  |  | **10%** |
|  |  |  |  |  |  |  |  |  |  |  |  |  |  |  |  |  |  |  |  |  |  |  |  |
| Appreciate the  uniqueness  inter-dependence and  universality of  man | The various explanations of the origin of man namely: religious, mythical and scientific. |  |  |  |  |  | X |  |  |  |  |  |  |  |  |  |  |  |  |  |  |  | **5%** |
|  | The beginning of man from Apes to homo-sapiens |  |  |  |  |  | X |  |  |  |  |  |  |  |  |  |  |  |  |  |  |  | **5%** |
|  | Harmonizing forces (tool making, Language, Social Organization and management of Man's Prolonged Childhood). |  |  |  |  |  | X |  |  |  |  |  |  |  |  |  |  |  |  |  |  |  | **5%** |
|  | The uniqueness of man |  |  |  |  |  | X |  |  |  |  |  |  |  |  |  |  |  |  |  |  |  | **5%** |
|  | The interdependence of man |  |  |  |  |  | X |  |  |  |  |  |  |  |  |  |  |  |  |  |  |  | **5%** |
|  | Race and Racism |  |  |  |  |  | X |  |  |  |  |  |  |  |  |  |  |  |  |  |  |  | **5%** |
|  | Humanity universality |  |  |  |  |  | X |  |  |  |  |  |  |  |  |  |  |  |  |  |  |  | **5%** |
|  |  |  |  |  |  |  |  |  |  |  |  |  |  |  |  |  |  |  |  |  |  |  |  |
| Know the  dynamics of  economic activities and demonstrate how they can contribute their quota to a stable  economy | Man's basic economic problems, Scarcity and choices |  |  |  |  |  |  | X |  |  |  |  |  |  |  |  |  |  |  |  |  |  | **5%** |
|  | Factors of production |  |  |  |  |  |  | X |  |  |  |  |  |  |  |  |  |  |  |  |  |  | **5%** |
|  | Man's reactions to supply and demand of goods and services. |  |  |  |  |  |  | X |  |  |  |  |  |  |  |  |  |  |  |  |  |  | **5%** |
|  | Production systems: primary, secondary and tertiary |  |  |  |  |  |  | X |  |  |  |  |  |  |  |  |  |  |  |  |  |  | **5%** |
|  | Sources of government revenue in Nigeria |  |  |  |  |  |  | X |  | X |  |  |  |  |  |  |  |  |  |  |  |  | **10%** |
|  | Economic problems: inflation, unemployment, poverty and poverty alleviation programmes. |  |  |  |  |  |  | X |  |  |  |  |  |  |  |  |  |  |  |  |  |  | **5%** |
|  |  |  |  |  |  |  |  |  |  |  |  |  |  |  |  |  |  |  |  |  |  |  |  |
| Comprehend the  relevance of  government in  the society and  the need to  participate. | Concepts & role of government in society |  |  |  |  |  |  |  | X | X |  |  |  |  |  |  |  |  |  |  |  |  | **10%** |
|  | Power & Authority |  |  |  |  |  |  |  | X |  |  |  |  |  |  |  |  |  |  |  |  |  | **5%** |
|  | Traditional forms government: Family, Clan, Village, Town, Empire etc | X |  |  |  |  |  |  | X |  |  |  |  |  |  |  |  |  |  |  |  |  | **10%** |
|  | Modern form of government: Democracy, Autocracy Monarchy, & the Military. |  |  |  |  |  |  |  | X | X |  |  |  |  |  |  |  |  |  |  |  |  | **10%** |
|  | Organs of government: Executive, Legislative, Judiciary and Press |  |  |  |  |  |  |  | X | X |  |  |  |  |  |  |  |  |  |  |  |  | **10%** |
|  | Tiers of government in Nigeria: Local. State and Federal emphasizing their structure functions. |  |  |  |  |  |  |  | X | X |  |  |  |  |  |  |  |  |  |  |  |  | **10%** |
|  |  |  |  |  |  |  |  |  |  |  |  |  |  |  |  |  |  |  |  |  |  |  |  |
| Demonstrate their awareness of the rule of law  and how it  relates to political issues. | Nigerian Political Life |  |  |  |  |  |  |  |  | X |  |  |  |  |  |  |  |  |  |  |  |  | **5%** |
|  | The Concepts of Nation, State and Country |  |  |  |  | X |  |  |  | X |  |  |  |  |  |  |  |  |  |  |  |  | **10%** |
|  | Nationalist movements and political parties before independence |  |  |  |  |  |  |  |  | X |  |  |  |  |  |  |  |  |  |  |  |  | **5%** |
|  | Independence, the Republics and the political parties |  |  |  |  |  |  |  |  | X |  |  |  |  |  |  |  |  |  |  |  |  | **5%** |
|  | Military rule in Nigeria |  |  |  |  |  |  |  | X | X |  |  |  |  |  |  |  |  |  |  |  |  | **10%** |
|  | Political Issues (Population Size, Power sharing/shift, Revenue allocation, Resource control etc) |  |  |  |  |  |  | X |  | X |  |  |  |  |  |  |  |  |  |  |  |  | **10%** |
|  | Constitutions (meaning, purpose and types) |  |  |  |  | X |  |  |  | X |  |  |  |  |  |  |  |  |  |  |  |  | **10%** |
|  | Constitutional developments in Nigeria since 1914 |  |  |  |  | X |  |  |  | X |  |  |  |  |  |  |  |  |  |  |  |  | **10%** |
|  | General provision of the current Nigeria constitution (Fundamental objectives & directive principles of state policy, citizenship, Fundamental Human Rights, Aims of government, FCT and General supplementary provision). |  |  |  |  | X |  |  | X | X |  |  |  |  | X |  | X |  |  |  |  |  | 24% |
|  |  |  |  |  |  |  |  |  |  |  |  |  |  |  |  |  |  |  |  |  |  |  |  |
| Demonstrate methods & techniques  necessary for the effective teaching and learning of SOS for Basic 7 – 9. | Dramatic representation |  |  |  | X |  |  |  |  |  | X |  |  |  |  |  |  |  | X |  |  |  | **14%** |
|  | Discussion |  |  |  | X |  |  |  |  |  | X |  |  |  |  |  |  |  | X |  |  |  | **14%** |
|  | Creative activities |  |  |  | X |  |  |  |  |  | X |  |  |  |  |  |  |  | X |  |  |  | **14%** |
|  | Simulation |  |  |  | X |  |  |  |  |  | X |  |  |  |  |  |  |  | X |  |  |  | **14%** |
|  | Problem solving |  |  |  | X |  |  |  |  |  | X |  |  |  |  |  |  |  | X |  |  |  | **14%** |
|  | Questioning technique |  |  |  | X |  |  |  |  |  | X |  |  |  |  |  |  |  | X |  |  |  | **14%** |
|  | Concept mapping etc. |  |  |  | X |  |  |  |  |  | X |  |  |  |  |  |  |  | X |  |  |  | **14%** |
|  |  |  |  |  |  |  |  |  |  |  |  |  |  |  |  |  |  |  |  |  |  |  |  |
| Students should be able to carry  out effective  research  work in Social  Studies | Concept and content of research: Types of research |  |  |  |  |  |  |  |  |  |  | X |  |  |  |  |  | X | X |  |  |  | **14%** |
|  | Concept and content of research: Choice of research topic |  |  |  |  |  |  |  |  |  |  | X |  |  |  |  |  |  |  |  |  |  | **5%** |
|  | Concept and content of research: Purpose /objectives of research. |  |  |  |  |  |  |  |  |  |  | X |  |  |  |  |  |  |  |  |  |  | **5%** |
|  | Review of relevant literature |  |  |  |  |  |  |  |  |  |  | X |  |  |  |  |  |  |  |  |  |  | **5%** |
|  | Research methodology (Research Design): Stating research problem |  |  |  |  |  |  |  |  |  |  | X |  |  |  |  |  |  |  |  |  |  | **5%** |
|  | Research methodology (Research Design): Choice of population |  |  |  |  |  |  |  |  |  |  | X |  |  |  |  |  |  |  |  |  |  | **5%** |
|  | Research methodology (Research Design): Sampling and sampling techniques |  |  |  |  |  |  |  |  |  |  | X |  |  |  |  |  |  |  |  |  |  | **5%** |
|  | Research methodology (Research Design): Hypothesizing |  |  |  |  |  |  |  |  |  |  | X |  |  |  |  |  |  |  |  |  |  | **5%** |
|  | Data collection techniques: Observation, interview, document, questionnaire etc |  |  |  |  |  |  |  |  |  |  | X |  |  |  |  |  |  |  |  |  |  | **5%** |
|  | Data collection techniques: Organization and presentation of data and statistical representation |  |  |  |  |  |  |  |  |  |  | X |  |  |  |  |  |  |  |  |  |  | **5%** |
|  | Appendices |  |  |  |  |  |  |  |  |  |  | X |  |  |  |  |  |  |  |  |  |  | **5%** |
|  | Bibliography and References |  |  |  |  |  |  |  |  |  |  | X |  |  |  |  |  |  |  |  |  |  | **5%** |
|  | Statistic (s): Meaning , types and uses of statistics |  |  |  |  |  |  |  |  |  |  | X |  |  |  |  |  |  |  |  |  |  | **5%** |
|  | Descriptive statistics: Measure of central tendency. |  |  |  |  |  |  |  |  |  |  | X |  |  |  |  |  |  |  |  |  |  | **5%** |
|  | Descriptive statistics: Measure of variability. |  |  |  |  |  |  |  |  |  |  | X |  |  |  |  |  |  |  |  |  |  | **5%** |
|  | Inferential statistics: Parametric |  |  |  |  |  |  |  |  |  |  | X |  |  |  |  |  |  |  |  |  |  | **5%** |
|  | Inferential statistics: Non-parametric. |  |  |  |  |  |  |  |  |  |  | X |  |  |  |  |  |  |  |  |  |  | **5%** |
|  |  |  |  |  |  |  |  |  |  |  |  |  |  |  |  |  |  |  |  |  |  |  |  |
| Write a study-  report on undertaking field  exercise and  develop in learners skills of  data collection | Students will be out for one to four days of studying both physical and social phenomenon, human activities in terms of housing, occupational practices, dressing, culture etc. |  |  |  |  |  |  |  |  |  |  |  | X |  |  |  |  |  |  |  |  |  | **5%** |
|  |  |  |  |  |  |  |  |  |  |  |  |  |  |  |  |  |  |  |  |  |  |  |  |
| Appraise and  address  problems of  National  Development. | National & concepts of national development. |  |  |  |  |  |  |  |  |  |  |  |  | X |  |  |  | X |  |  |  |  | **10%** |
|  | Meaning, nature and relationship between modernization & national development. |  |  |  |  |  |  |  |  |  |  |  |  | X |  |  |  |  |  |  |  |  | **5%** |
|  | Dimensions of national development (economic development, political development, social development, legal development, educational development, technology & health development etc) |  |  |  |  |  |  |  |  |  |  |  |  | X |  |  |  |  |  |  |  |  | **5%** |
|  | Problems of national development (poor data base, corruption, poor plan implementation, external manipulations and illiteracy etc) |  |  |  |  |  |  |  |  |  |  |  |  | X |  |  |  |  |  |  |  |  | **5%** |
|  | Factors and processes of modernization |  |  |  |  |  |  |  |  |  |  |  |  | X |  |  |  |  |  |  |  |  | **5%** |
|  | Aspects of modernization (Population, urbanization, education, science & technology, socio-cultural, political & economic). |  |  |  |  |  |  |  |  |  |  |  |  | X |  | X |  |  | X |  |  |  | 29% |
|  |  |  |  |  |  |  |  |  |  |  |  |  |  |  |  |  |  |  |  |  |  |  |  |
| Demonstrate  positive  qualities of good citizenship | Concept of socialization |  |  |  |  |  |  |  |  |  |  |  |  |  | X |  |  |  |  |  |  |  | **5%** |
|  | Types of socialization (Primary, secondary, adult) |  |  |  |  |  |  |  |  |  |  |  |  |  | X |  |  |  |  |  |  |  | **5%** |
|  | Agents of socialization (Family, peer group, school, mass media, church, mosque etc.) | X | X |  |  |  |  |  | X |  |  |  |  |  | X | X |  |  | X |  |  |  | 29% |
|  | Processes of socialization |  |  |  |  |  |  |  |  |  |  |  |  |  | X |  |  |  |  |  |  |  | **5%** |
|  | Political socialization & mass mobilization (MAMSER, NOA, etc |  |  |  |  |  |  |  |  |  |  |  |  |  | X |  |  |  |  |  |  |  | **5%** |
|  | Problems of socialization |  |  |  |  |  |  |  |  |  |  |  |  |  | X |  |  |  |  |  |  |  | **5%** |
|  | The role of SOS in the socialization & production of good citizens |  |  |  |  |  |  |  |  |  |  |  |  |  | X |  |  |  |  |  |  |  | **5%** |
|  | The concepts of citizen & citizenship education |  |  |  |  |  |  |  |  | X |  |  |  |  | X |  |  |  |  |  |  |  | **10%** |
|  | Types of citizenship (single & dual) |  |  |  |  |  |  |  |  | X |  |  |  |  | X |  |  |  |  |  |  |  | **10%** |
|  | Citizenship acquisition in Nigeria (By birth, by registration & by national naturalization) |  |  |  |  |  |  |  |  | X |  |  |  |  | X |  |  |  |  |  |  |  | **10%** |
|  | Renunciation and denial of citizenship |  |  |  |  |  |  |  |  | X |  |  |  |  | X |  |  |  |  |  |  |  | **10%** |
|  | Qualities and duties of a good citizen |  |  |  |  |  |  |  |  | X |  |  |  |  | X |  |  |  |  |  |  |  | **10%** |
|  | Fundamental Human Rights |  |  |  |  |  |  |  |  | X |  |  |  |  | X |  |  |  |  |  |  |  | **10%** |
|  | Lawful denial of fundamental human rights |  |  |  |  |  |  |  |  | X |  |  |  |  | X |  |  |  |  |  |  |  | **10%** |
|  | Violation & protection of Human Rights |  |  |  |  |  |  |  |  | X |  |  |  |  | X |  |  |  |  |  |  |  | **10%** |
|  | Ways in which Human Rights are violated |  |  |  |  |  |  |  |  | X |  |  |  |  | X |  |  |  |  |  |  |  | **10%** |
|  | Ways of protecting Human Rights |  |  |  |  |  |  |  |  | X |  |  |  |  | X |  |  |  |  |  |  |  | **10%** |
|  |  |  |  |  |  |  |  |  |  |  |  |  |  |  |  |  |  |  |  |  |  |  |  |
| Appraise the  structure,  functions  and problems of  providing social  services in  Nigeria | Social administration and social policies defined |  | X |  |  |  |  |  |  |  |  |  |  |  |  | X |  |  |  |  | X |  | **14%** |
|  | Educational institutions: Structures and functions in Nigeria |  |  |  |  |  |  |  |  |  |  |  |  |  |  | X |  |  |  |  | X |  | **10%** |
|  | Health institutions: Structure and functions, National AIDS/STI Control Programmes in Nigeria (NASCP) | X |  |  |  |  |  |  |  |  |  |  |  |  |  | X |  |  | X |  | X |  | **19%** |
|  | Housing Policy |  |  |  |  |  |  |  |  |  |  |  |  |  |  | X |  |  |  |  |  |  | **5%** |
|  | Other services and utilities: Fire, Prison, Postal, Old age pension, Nigeria Police Force, Water Supply, Electricity, Transport, Communication. |  |  |  |  |  |  |  |  |  |  |  |  |  |  | X | X | X |  |  |  |  | **14%** |
|  | Attitude to public utilities |  |  |  |  |  |  |  |  |  |  |  |  |  |  | X |  |  |  |  |  |  | **5%** |
|  | Population pressure on social services in Nigeria. | X |  |  |  | X |  |  |  |  |  |  |  | X |  | X |  |  |  |  |  |  | **19%** |
|  | The concept of change |  |  |  |  |  |  |  |  |  |  |  |  |  |  | X |  |  |  |  |  |  | **5%** |
|  | Theories of change |  |  |  |  |  |  |  |  |  |  |  |  |  |  | X |  |  |  |  |  |  | **5%** |
|  | Types of change |  |  |  |  |  |  |  |  |  |  |  |  |  |  | X |  |  |  |  |  |  | **5%** |
|  | Factors and processes of change |  |  |  |  |  |  |  |  |  |  |  |  |  |  | X |  |  |  |  |  |  | **5%** |
|  | Changes in Nigeria before and after 1960 in demographic, economic. Socio-cultural and political system. |  |  |  |  |  |  |  |  |  |  |  |  |  |  | X |  |  |  |  |  |  | **5%** |
|  | Change and its effects on the individual and the family in Nigeria. |  | X |  |  |  |  |  |  |  |  |  |  |  | X | X |  |  | X |  |  |  | **19%** |
|  |  |  |  |  |  |  |  |  |  |  |  |  |  |  |  |  |  |  |  |  |  |  |  |
| Having an insight  into who is responsible for  promulgating and executing the laws of the  society. | Introducing the learner to the constitutions of the Nigerian government, colonial heritage, traditions and sharing. |  | X |  |  |  |  |  |  |  |  |  |  |  |  |  | X |  |  |  |  |  | **10%** |
|  | The process of law making in Nigeria |  |  |  |  |  |  |  |  |  |  |  |  |  |  |  | X |  |  |  |  |  | **5%** |
|  | Litigations, criminal and civil cases |  |  |  |  |  |  |  |  |  |  |  |  |  |  |  | X |  |  |  |  |  | **5%** |
|  | Administration of justice: The function of the police, courts and law and prisons services. |  |  |  |  |  |  |  |  |  |  |  |  |  |  | X | X |  |  |  |  |  | **10%** |
|  | Critical look at the role of the Judiciary in the implementation of the law e.g. the hierarchy of courts, personal and independence. |  |  |  |  |  |  |  |  | X |  |  |  |  |  |  | X |  |  |  |  |  | **10%** |
|  | A detail study of "You and the Law". |  |  |  |  |  |  |  |  |  |  |  |  |  |  |  | X |  |  |  |  |  | **5%** |
|  |  |  |  |  |  |  |  |  |  |  |  |  |  |  |  |  |  |  |  |  |  |  |  |
| Appraise the  problems and  prospects of  transport and communication | The differences between transportation and communication |  |  |  |  |  |  |  |  |  |  |  |  |  |  | X |  | X |  |  |  |  | **10%** |
|  | Traditional and modern means of transportation: Advantages and problems. |  |  |  |  |  |  |  |  |  |  |  |  |  |  |  |  | X |  |  |  |  | **5%** |
|  | Traditional and modern means of communication: Advantages and problems. Email, fax, telex, radio and internet. Practical application should be demonstrated to students. |  |  |  |  |  |  |  |  |  |  |  |  |  |  |  |  | X |  |  |  |  | **5%** |
|  | The role of transportation and communication on national development. |  |  |  |  |  |  |  |  |  |  |  |  | X |  | X |  | X |  |  |  |  | **14%** |
|  | The mass media: What is mass media, their role in national development, Problems etc.? |  |  |  |  |  |  |  |  |  |  |  |  | X |  | X |  | X |  |  |  |  | **14%** |
|  | Students should develop case studies materials on any mass media of their choice (It should form part of CA). |  |  |  | X |  |  |  |  |  |  | X |  |  |  |  |  | X | X |  |  |  | **19%** |
|  |  |  |  |  |  |  |  |  |  |  |  |  |  |  |  |  |  |  |  |  |  |  |  |
| Demonstrate  Positive  attitudes towards family  life | The concept of population |  |  |  |  | X |  |  |  |  |  |  |  | X |  | X |  |  | X |  |  |  | **19%** |
|  | The concept of family life |  | X |  |  |  |  |  |  |  |  |  |  |  | X |  |  |  | X |  |  |  | **14%** |
|  | The family life education |  | X |  |  |  |  |  |  |  |  |  |  |  | X |  |  |  | X |  |  |  | **14%** |
|  | The objective of population education |  |  |  |  | X |  |  |  |  |  |  |  | X |  | X |  |  | X |  |  |  | **19%** |
|  | The objective of family life education |  | X |  |  |  |  |  |  |  |  |  |  |  | X |  |  |  | X |  |  |  | **14%** |
|  | Gender issues and family life education. |  | X |  |  |  |  |  |  |  |  |  |  |  | X |  |  |  | X |  |  |  | **14%** |
|  | Family size and welfare |  | X |  |  |  |  |  |  |  |  |  |  |  | X |  |  |  | X |  |  |  | **14%** |
|  | The roles of members of the family |  | X |  |  |  |  |  |  |  |  |  |  |  | X |  |  |  | X |  |  |  | **14%** |
|  | The responsibility of parenthood |  |  |  |  |  |  |  |  |  |  |  |  |  |  |  |  |  | X |  |  |  | **5%** |
|  | Population data (Census & Vital registration) |  |  |  |  | X |  |  |  |  |  |  |  | X |  | X |  |  | X |  |  |  | **19%** |
|  | Population distribution in Nigeria and Africa |  |  |  |  |  |  |  |  |  |  |  |  | X |  | X |  |  | X |  |  |  | **14%** |
|  | The relationship between SOS and Population. Family Life and AIDS Education | X | X |  |  | X |  |  |  |  |  |  |  | X |  | X |  |  | X |  |  |  | 29% |
|  | National Population Policy (NPP) |  |  |  |  | X |  |  |  |  |  |  |  | X |  | X |  |  | X |  |  |  | **19%** |
|  | Population dynamics: Growth, decline and structure; and their socio-economic implication |  |  |  |  | X |  |  |  |  |  |  |  | X |  | X |  |  | X |  |  |  | **19%** |
|  | Methods of teaching Population/Family Life Education |  | X |  | X | X |  |  |  |  | X |  |  | X | X | X |  | X | X |  |  |  | 43% |
|  |  |  |  |  |  |  |  |  |  |  |  |  |  |  |  |  |  |  |  |  |  |  |  |
| Appraise the role of Nigeria in the  International  Community | The concept of International Relations |  |  |  |  |  |  |  |  |  |  |  |  |  |  |  |  |  |  | X |  |  | **5%** |
|  | Nigerian Foreign Policies (Principles & Policies) |  |  |  |  |  |  |  |  |  |  |  |  |  |  |  |  |  |  | X |  |  | **5%** |
|  | Nigeria & ECOWAS (Formation, functioning & problems) |  |  |  |  |  |  |  |  |  |  |  |  |  |  |  |  |  |  | X |  |  | **5%** |
|  | Man in International Community |  |  |  |  |  |  |  |  |  |  |  |  |  |  |  |  |  |  | X |  |  | **5%** |
|  | Word Tension: Causes and solutions (Games, conferences & membership etc) |  |  |  |  |  |  |  |  |  |  |  |  |  |  |  |  |  |  | X |  |  | **5%** |
|  | Nigeria in the Commonwealth |  |  |  |  |  |  |  |  |  |  |  |  |  |  |  |  |  |  | X |  |  | **5%** |
|  | Nigeria in OPEC |  |  |  |  |  |  |  |  |  |  |  |  |  |  |  |  |  |  | X |  |  | **5%** |
|  | Nigeria in the UNO (Contributions, benefits & problems) |  |  |  |  |  |  |  |  |  |  |  |  |  |  |  |  |  |  | X |  |  | **5%** |
|  | Nigeria in Africa Union (AU) |  |  |  |  |  |  |  |  |  |  |  |  |  |  |  |  |  |  | X |  |  | **5%** |
|  |  |  |  |  |  |  |  |  |  |  |  |  |  |  |  |  |  |  |  |  |  |  |  |
| Proffer possible  solutions to the  problems affecting social institutions in  Nigeria | The concept of social institution |  | X |  |  |  |  |  |  |  |  |  |  |  |  | X |  |  |  |  | X |  | **14%** |
|  | Structure and functions of different social institutions such as legal, political, economic, religious, educational, health institutions etc, in Nigeria. |  | X |  |  |  |  |  |  |  |  |  |  |  |  | X |  |  |  |  | X |  | **14%** |
|  | Problems of social institutions in Nigeria. |  | X |  |  |  |  |  |  |  |  |  |  |  |  | X |  |  |  |  | X |  | **14%** |
|  | Religion in society |  | X |  |  |  |  |  |  |  |  |  |  |  |  |  |  |  |  |  | X |  | **10%** |
|  | Religion in Nigeria |  | X |  |  |  |  |  |  |  |  |  |  |  |  |  |  |  |  |  | X |  | **10%** |
|  | Religion and morality |  | X |  |  |  |  |  |  |  |  |  |  |  |  |  |  |  |  |  | X |  | **10%** |
|  | Religion and politics |  | X |  |  |  |  |  |  |  |  |  |  |  |  |  |  |  |  |  | X |  | **10%** |
|  | Conflict and tolerance in Nigeria. |  |  |  |  |  |  |  |  |  |  |  |  |  |  |  |  |  |  |  | X |  | **5%** |
|  |  |  |  |  |  |  |  |  |  |  |  |  |  |  |  |  |  |  |  |  |  |  |  |
| Develop awareness and appreciation of  the changes globalization has  be on the Nigerian society. | The concept of globalization |  |  |  |  |  |  |  |  |  |  |  |  |  |  |  |  |  |  |  |  | X | **5%** |
|  | Historical antecedents (Colonialism, Imperialism, Europeanization, Westernization, Americanization etc.) |  |  |  |  |  |  |  |  |  |  |  |  |  |  |  |  |  |  |  |  | X | **5%** |
|  | Who is globalizing, and what is being globalized? |  |  |  |  |  |  |  |  |  |  |  |  |  |  |  |  |  |  |  |  | X | **5%** |
|  | Who is globalizing, and what cannot be globalized? |  |  |  |  |  |  |  |  |  |  |  |  |  |  |  |  |  |  |  |  | X | **5%** |
|  | Impact of globalization on the South (i.e. developing and underdeveloped countries, including Nigeria. |  |  |  |  |  |  |  |  |  |  |  |  |  |  |  |  |  |  |  |  | X | **5%** |
|  | What can Nigeria globalize? How? (i.e. Nigeria and the globalization process). |  |  |  |  |  |  |  |  |  |  |  |  |  |  |  |  |  |  |  |  | X | **5%** |
|  |  |  |  |  |  |  |  |  |  |  |  |  |  |  |  |  |  |  |  |  |  |  |  |

Course codes in cells with green backgrounds are required courses and yellow background denote elective courses in Social Studies Department. There are 21 total NCE Social Studies courses. Cells highlighted red in % indicates possible gaps in competency coverage. There are no possible redundancies in competency coverage.
